# Susceptibility of anurans, lizards, and fish to infection with *Dracunculus* species larvae and implications for their roles as paratenic hosts

**DOI:** 10.1038/s41598-021-91122-5

**Published:** 2021-06-03

**Authors:** Erin K. Box, Michael J. Yabsley, Kayla B. Garrett, Alec T. Thompson, Seth T. Wyckoff, Christopher A. Cleveland

**Affiliations:** 1 Southeastern Cooperative Wildlife Disease Study, Department of Population Health, College of Veterinary Medicine, University of Georgia, 589 D. W. Brooks Drive, Athens, GA 30602 USA; 2 Warnell School of Forestry and Natural Resources, University of Georgia, Athens, GA USA

**Keywords:** Ecology, Population dynamics

## Abstract

*Dracunculus* spp. are parasitic nematodes that infect numerous species of mammals and reptiles. The life cycles of *Dracunculus* species are complex, and unknowns remain regarding the role of paratenic and transport hosts in transmission to definitive hosts. We had two primary objectives: to assess the susceptibility of several species of anurans, lizards, and fish as paratenic hosts for *Dracunculus* species, and to determine the long-term persistence of *Dracunculus* infections in African clawed frogs (*Xenopus laevis*). Animals were orally exposed to copepods infected with infectious third-stage larvae (L3s) of either *Dracunculus insignis* or *D. medinensis*. *Dracunculus* L3s were recovered from four anuran species, two lizard species, and one fish species, demonstrating that *Dracunculus* can infect tissues of a diversity of species. In long-term persistence trials, *D. medinensis* L3s were recovered from African clawed frogs tissues up to 58 days post-infection, and *D. insignis* L3s were recovered up to 244 days post-infection. Our findings regarding the susceptibility of novel species of frogs, lizards, and fish to infection with *Dracunculus* nematodes, and long-term persistence of L3s in paratenic hosts, address pressing knowledge gaps regarding *Dracunculus* infection in paratenic hosts and may guide future research regarding the transmission of *Dracunculus* to definitive mammalian hosts.

## Introduction

*Dracunculus* nematodes are endoparasites which infect, as definitive hosts, a high diversity of mammal and reptile species on multiple continents^1^. The most well-known species in this genus is *Dracunculus medinensis*, or the human Guinea worm, for which humans are considered the primary definitive host^2^. This parasite has been targeted for eradication by a global Guinea Worm Eradication Program (GWEP), which has succeeded in decreasing human case numbers by over 99.9%^3^. In recent decades, an increasing number of *D. medinensis* infections have been reported from domestic dogs (*Canis lupus familiaris*), particularly in Chad, Africa, which poses a serious challenge for the GWEP^2,4^. There are also multiple species of *Dracunculus* that infect mammals in the Americas, including *Dracunculus insignis* which infects a wide range of mammals (e.g., raccoons [*Procyon lotor*], North American river otters [*Lontra canadensis*], Virginia opossums [*Didelphis virginianus*], domestic dogs, and cats [*Felis catus*])^1^.

In definitive hosts, the large, gravid female *Dracunculus* typically migrates to the distal extremities of the infected host where a blister is formed at the eventual site of emergence^5^. The anterior end of the worm emerges from the blister and, if the worm is submerged in water, hundreds of thousands of free-swimming, first-stage larvae (L1s) will be released^5^. Cyclopoid copepods (the intermediate host) must ingest these larvae for them to develop into third-stage larvae (L3s), which are infectious to subsequent paratenic or definitive hosts, within the copepod^5^. In the life cycle of *Dracunculus* spp., the term transport host is used separately from paratenic host and refers to hosts in which infected copepods are ingested, but larvae never leave the gastrointestinal tract. However, ingestion of these transport hosts while L3s are present in the gastrointestinal tract may result in infection (distinction from paratenic hosts is discussed in references)^6,7^. In humans, infection with *D. medinensis* classically occurs by drinking water contaminated with infected copepods^8^. Emergence of the female worm typically occurs one year after infection^5^.

While the life cycle of *D. medinensis* and its transmission to humans is relatively well studied and understood, there remain gaps in scientific knowledge regarding the primary mode of natural infection for other mammalian hosts. It is hypothesized that direct ingestion of copepods may not explain patterns of *D. medinensis* transmission seen in dogs in Sub-Saharan Africa, because, when drinking, many animals lap (e.g., dogs and opossums) or suck (e.g., many snake species) from the top of the water column^9–12^. Copepods are often found lower in the water column, especially when infected with *Dracunculus*^13,14^. For these reasons, it has been suggested that *Dracunculus* transmission to wildlife and domestic animal hosts (as well as, potentially, humans) may also occur through alternative infection routes (e.g., the use of a paratenic or transport host infected with L3s)^6,7^.

Fish, amphibians, and reptiles have been investigated for their potential as paratenic hosts of several *Dracunculus* species^6,15–17^. Previous work has demonstrated that laboratory infected anurans can act as paratenic hosts for *Dracunculus ophidensis* to snakes (common garter snakes [*Thamnophis sirtalis*] and northern water snakes [*Nerodia sipedon*]), *D. insignis* to raccoons and domestic ferrets (*Mustela putorius furo*), and *D. medinensis* to domestic ferrets^14–16^. *Dracunculus insignis* larvae have been previously recovered from infected amphibians up to 37 days post-exposure which was the latest time period the amphibians were necropsied^16^. *Dracunculus medinensis* L3s have been recovered from wild frogs (*Hoplobatrachus occipitalis* and *Phrynobatrachus francisci*) in Chad, Africa, and *D. insignis* L3s have been recovered from wild frogs (*Lithobates* [*Rana*] *catesbeiana* and *Lithobates* [*Rana*] *sphenocephalus*) in Georgia, USA^17–19^.

Tissues of wild-caught fish have been inspected for the presence of *Dracunculus* larvae, but no larvae have been recovered^17,19^. Fish have previously been experimentally inoculated with or fed *Dracunculus* L3s and *Dracunculus*-infected copepods; however, L3s have been recovered from only a small number of fish and the number of larvae recovered from each animal was low, suggesting that they are not likely common paratenic hosts^6^. However, some fish species have been shown to serve as short-term transport hosts for *Dracunculus* species, successfully transmitting *D. insignis* and *D. medinensis* to ferrets^7^.

Historically, subcutaneous worms detected in Nile monitor lizards (*Varanus niloticus*) have been reported to be adult *Dracunculus*, although few morphologic features were noted and none have been genetically confirmed to be *Dracunculus*^20^. More recently, suspect subcutaneous nematodes found in Nile monitor lizards in Chad were determined not to be *Dracunculus* species, but similar to *Ochoterenella* species^17^. Although these investigations have predominately focused on adult subcutaneous worms, the possibility remains that Nile monitors may be potential paratenic hosts, susceptible to infection with *Dracunculus* sp. larvae that do not mature into adult worms.

The objectives of this study were to investigate the susceptibility of several anuran, lizard, and fish species to infection with *Dracunculus* spp. L3s and to determine the long-term persistence of *Dracunculus* larvae in anurans. We hypothesized that anurans would become most readily infected and that lizards and fish would develop few, if any, infections. We also hypothesized that larvae would persist at least several weeks, likely longer, within infected paratenic hosts. The insight gained from this work will help to better understand the role that these animals may play as paratenic hosts for *Dracunculus* transmission to domestic animals and wildlife hosts.

## Results

### Infection of potential paratenic hosts with *Dracunculus*-infected copepods

#### Anurans

Among the anuran species tested, four of five species (African clawed frogs [*Xenopus laevis*], American toad [*Anaxyrus* (*Bufo*) *americanus*], Cope’s gray treefrog [*Hyla chrysoscelis*], and southern leopard frog [*Lithobates* (*Rana*) *sphenocephalus*]) developed infections with *Dracunculus*. *Dracunculus insignis* larvae were recovered from 10 of 22 (45%) anurans that were exposed by group batch with 20 copepods offered per individual. All recovered larvae (n = 1–14) were from muscle tissue (Table 1). No larvae were recovered from anurans that were exposed by group batch methods with only 10 copepods offered per individual (Table 1).

**Table 1:** Methods and results of paratenic host experimental infections with *Dracunculus insignis* or *Dracunculus medinensis* third-stage larvae (L3s) via ingestion of infected copepods. All larvae recovered remained L3s.

Host species	*Dracunculus* species	Age group	No. hosts	No. copepods per host	Exposure type	Days from exposure to necropsy	Infection status (No. positive)	No. larvae recovered per host
**ANURANS**								
African clawed frog (*Xenopus laevis*)	*D. insignis*	Tadpole	5	10	GB	11	NEG	
	*D. insignis*	Tadpole	8	10	GB	29	NEG	
	*D. insignis*	Tadpole	6	10	GB	29	NEG	
	*D. insignis*	Adult	3	20	GB	99	**POS** (1)	2
	*D. insignis*	Adult	2	10	GB	140	NEG	
Southern leopard frog (*Lithobates* [*Rana*] *sphenocephalus*)	*D. insignis*	Tadpole	2	10	GB	9	NEG	
	*D. insignis*	Tadpole	2	10	GB	11	NEG	
	*D. insignis*	Tadpole	3	10	GB	29	NEG	
	*D. insignis*	Tadpole	3	10	GB	31	NEG	
	*D. insignis*	tp➝froglet*	4	20	GB	44	**POS** (2)	1 & 1
	*D. insignis*	tp➝froglet*	2	20	GB	50	NEG	
	*D. insignis*	Tadpole	6	20	GB	58	**POS** (4)	3, 4, 8 & 8
	*D. insignis*	Tadpole	2	20	GB	70	**POS** (2)	1 & 1
	*D. insignis*	Tadpole	1	20	GB	76	NEG	
	*D. insignis*	Tadpole	1	20	GB	86	NEG	
	*D. insignis*	Tadpole	1	20	GB	97	**POS** (1)	14
American bullfrog (*Lithobates* [*Rana*] *catesbeianus*)	*D. insignis*	Tadpole	6	10	GB	16	NEG	
Cope’s gray treefrog (*Hyla chrysoscelis*)	*D. medinensis*	Adult	2	20	PO	6	**POS** (1)	15
	*D. insignis*	Tadpole	2	20	GB	51	NEG	
American toad (*Anaxyrus* [*Bufo*] *americanus*)	*D. medinensis*	Adult	5	20	PO	6	**POS** (2)	1 & 1
**LIZARDS**								
Nile monitor (*Varanus niloticus*)	*D. insignis*	Juvenile	1	25	PO	12	NEG	
	*D. insignis*	Juvenile	2	25	PO	13	**POS** (1)	1
	*D. insignis*	Juvenile	1	25	PO	14	**POS** (1)	1
	*D. insignis*	Juvenile	1	25	PO	15	NEG	
Green anole (*Anolis carolinensis*)	*D. insignis & D. medinensis*	Adult	1	25 (23 *D. i. &* 2 *D. m.*)	PO	6	**POS** (1)	4
**FISH**								
Bichir (*Polypterus* sp.)	*D. medinensis*	Juvenile	4	20	PO (2)/ GB (2)	10	NEG	
Featherfin catfish (*Synodontis eupterus*)	*D. medinensis*	Juvenile	4	20	GB	10	**POS** (3)	2, 3 & 6

*D. i.* = *Dracunculus insignis*; *D. m.* = *D. medinensis*; GB = group batch; PO = by mouth; tp = tadpole

*Indicates metamorphosis occurred between infection and necropsy.

*Dracunculus medinensis* larvae were recovered from three out of seven (43%) of the anurans exposed to 20 infected copepods (Table 1). Larvae from *D. medinensis-*infected anurans were all recovered at six days post-inoculation (DPI) (Table 1). All recovered *D. medinensis* larvae were from muscle tissue, except one, which was recovered from the viscera of an American toad.

#### Lizards

A single *D. insignis* larva was recovered each from two of five (40%) Nile monitor lizards (Table 1). A single *D. insignis* larva was recovered from gastrointestinal or visceral tissue of one monitor lizard at DPI 13 and a single *D. insignis* larva was recovered from the muscle from the other monitor lizard on DPI 14. Four *D*. *insignis* and/or *D. medinensis* larvae were recovered from the single exposed green anole (*Anolis carolinensis*) at DPI 6 (Table 1). These four *Dracunculus* sp. larvae were recovered from the abdomen (n = 2), tail or legs (n = 1), and viscera (n = 1) of the anole.

#### Fish

*Dracunculus medinensis* larvae were recovered from the muscle of three of four (75%) featherfin catfish (*Synodontis eupterus*) at 10 DPI (Table 1). All four exposed bichir (*Polypterus* sp.) were negative (Table 1).

### Ability of *Dracunculus* L3s from a paratenic host to infect another paratenic host

No larvae were recovered from the African clawed frogs that were exposed to *D. medinensis* larvae recovered from previous paratenic hosts.

### Persistence of *Dracunculus* in paratenic hosts

*Dracunculus insignis* larvae (n = 1–8) were recovered from adult African clawed frogs at approximately three, four, six, and eight (maximum length of time tested) months post-infection. A single *D. medinensis* larva was recovered from an adult African clawed frog at approximately two months post-infection, but not beyond. All larvae remained L3s (Table 2).

**Table 2:** Results of experimental infection and long-term persistence trials of African clawed frogs (*Xenopus laevis*) exposed by mouth (PO) to 50 copepods infected with third-stage *Dracunculus insignis* or *Dracunculus medinensis* larvae. All larvae recovered remained L3s.

*Dracunculus* species	No. hosts	Approx. months from exposure to necropsy	Days from exposure to necropsy	Infection status (no. positive)	No. larvae recovered per host
*D. insignis*	10	3	99	**POS** (1)	1
*D. insignis*	5	4	121	**POS** (1)	6
*D. insignis*	5	5	141	NEG	
*D. insignis*	3	6	188	**POS** (2)	1 & 1
*D. insignis*	5	8	244	**POS** (1)	8
*D. medinensis*	1	2	58	**POS** (1)	1
*D. medinensis*	1	4	115	NEG	
*D. medinensis*	1	4	125	NEG	

## Discussion

This study demonstrated that several anuran genera (*Xenopus*, *Lithobates* [*Rana*], *Hyla*, and *Anaxyrus* [*Bufo*]), as well as Nile monitor lizards, green anoles, and featherfin catfish, are susceptible to infection with *D. insignis* and/or *D. medinensis* L3s. We also found that *D. insignis* and *D. medinensis* larvae can persist in anuran tissues for at least eight and two months, respectively, although the number of L3s recovered from each infected animal was generally low. Regardless, these data show that these animals could serve as paratenic hosts if they ingest infected copepods in nature and are subsequently ingested by an appropriate definitive host.

We exposed animals using two different methods (group batch or by mouth [PO]), but aimed to primarily batch expose animals as that better mimics natural exposure. A few anuran species (i.e., American toads, Cope’s gray treefrogs, and adult African clawed frogs) were exposed to *D. medinensis*-infected copepods PO, as they had metamorphosed into adults before *D. medinensis* larvae became available for use and would be unlikely to ingest all copepods autonomously. Our primary goal in this study was to determine susceptibility to *Dracunculus* infection.

Six anurans that were exposed as tadpoles underwent metamorphosis to froglets before being necropsied. *Dracunculus* L3s were recovered from two of these animals, supporting previous findings that *D. insignis* larvae can persist in anuran tissues through metamorphosis^14^. The persistence of larvae in the tissues through metamorphosis may facilitate *Dracunculus* transmission from aquatic to terrestrial food chains. This could be an important factor in transmission, as the majority of definitive hosts of *Dracunculus* nematodes are terrestrial. This study found that, in addition to *X. laevis* and *Lithobates* spp. (which have previously been infected with *Dracunculus* spp. larvae), *Anaxyrus* sp. and *Hyla* sp. can also become infected with *Dracunculus* L3s^14,21^. The infection of *Anaxyrus* sp. and *Hyla* sp. is particularly interesting, as members of these genera transition to a terrestrial or arboreal existence as adults, compared to *Xenopus* sp. and *Lithobates* spp. which remain completely or predominantly aquatic, even as adults. This transition to a terrestrial habitat could carry infectious larvae further from water sources, making them available to definitive hosts more widely across the landscape. However, the role of these animals in *Dracunculus* transmission would still depend on many other factors, including the natural history of these amphibian species, diets of definitive hosts, and how long *Dracunculus* L3s persist in paratenic hosts, as terrestrial anurans would be unlikely to acquire new infections after metamorphosis.

During a previous experimental study, *D. insignis* L3s persisted in amphibian paratenic hosts for up to 37 DPI, at which time the animals were necropsied^16^. In this long-term infection trial, we found that *D. insignis* larvae persisted for at least 244 days (approximately eight months), while *D. medinensis* larvae persisted for at least 58 days (approximately two months). These results demonstrate that infection of a paratenic host can extend the time that L3s may persist in the environment well beyond the lifespan of a copepod^21^. As we had a limited supply of *D. medinensis* L3s, we were unable to conduct sufficient trials to determine whether *D. insignis* may persist longer in paratenic hosts than *D. medinensis*. If this difference was found to exist, it could contribute to the higher proportion of wild-caught adult frogs found to be infected with *D. insignis* than with *D. medinensis* during field surveys^18,19^. Further testing with an increased sample size would be required to determine whether the persistence of larvae actually differs between *Dracunculus* species or paratenic host species.

No *Dracunculus* larvae were recovered from the two adult African clawed frogs that were fed *D. medinensis* L3s that had been recovered from other paratenic hosts. It is likely that our very small sample size (two animals) and the prolonged period before necropsy (4 months) explain these negative results. In our persistence trials, there was attrition over time so these animals should have been examined earlier after exposure. Future efforts to investigate transmission of *Dracunculus* between different paratenic hosts should use larger sample sizes and shorter infection periods. It would also be interesting to know if predatory animals, such as Nile monitor lizards, which can experimentally become infected with *Dracunculus* sp. larvae could become infected by ingesting other paratenic hosts.

Fish were investigated for their potential role in *Dracunculus* transmission, as many fish species consume copepods as part of a natural diet^25,26^. Despite this, *Dracunculus* larvae have not been recovered during multiple studies screening wild-caught fish^17,19^. *Dracunculus insignis* L3s have rarely been recovered from previous experimental trials with fish^16^. When larvae were recovered from fish, larval recovery rates were very low (0.6–2.0% recovery; 1–2 larvae per fish) and only 3/43 (7.0%) of the fish harbored *Dracunculus* larvae upon necropsy^16^. In a separate trial, fish experimentally functioned as short-term transport hosts of *D. medinensis* and *D. insignis* to infect domestic ferrets^7^. Our findings from this trial were surprising, as we recovered up to 6 *D. medinensis* L3s from the tissues of three out of four (75%) exposed featherfin catfish. This fish species is common in the Chari River Basin area in Chad, Africa where high numbers of *D. medinensis* infections are reported in domestic dogs living in fishing villages, and is consumed by both people and dogs^17^. Dogs in these villages often eat discarded small fish or fish viscera^4^. Although our sample size was small, our current findings are evidence that some fish species may be more capable of serving as paratenic hosts for *Dracunculus* than those that have been previously tested. This finding further supports the continuation of the screening of wild fish muscle tissues for *Dracunculus* larvae.

Lizards were included in this study because large, subcutaneous nematodes (believed to be *Dracunculus* sp.) were historically reported from Nile monitor lizards and these lizards are consumed by people^20,22^. However, a lack of contemporary reports and recent work in Chad, Africa, determining that large, subcutaneous nematodes recovered from wild Nile monitor lizards were not *Dracunculus* sp. but actually most similar to *Ochoterenella* sp., suggest that monitor lizards in this region are not definitive hosts for *D. medinensis*^17^. This current study confirms that Nile monitor and green anole lizards could become infected with *Dracunculus* larvae. As the diet of Nile monitor lizards can include amphibians and fish, were those prey to contain *Dracunculus* larvae, it is possible that monitors could serve as paratenic hosts, either by ingestion of larvae in fish intestines or in tissues of amphibians or fish, although these modes of transmission to paratenic hosts have not been confirmed^23,24^. It is unlikely that green anoles would become naturally infected with *Dracunculus* spp. due to their diet and primarily arboreal habitat; however, their infection demonstrates that multiple, distantly related lizard species are susceptible to experimental infection.

Although anoles were exposed to both *D. insignis* and *D. medinensis* larvae, it is most likely that the recovered larvae were *D. insignis*, as only two *D. medinensis*-infected copepods were administered (in addition to 23 *D. insignis*-infected copepods). Species identity of these larvae could not be confirmed, however, as *Dracunculus* larvae can only be identified to species using molecular diagnostic techniques, which would destroy the sample, and these larvae were used in an experimental infection trial after recovery. Exposure of a ferret PO to the four larvae recovered from this anole (as part of a separate study) did not yield an infection, which is unsurprising given the low dose of larvae used. A previous study has shown that as few as 10 *Dracunculus* larvae may lead to infection of a ferret when administered interperitoneally (IP) (which was a more effective infection route than PO inoculation), therefore, four larvae administered PO would be unlikely to yield infection of a ferret^27,28^.

In all trials, infection occurred only in those animals that were inoculated with or exposed to at least 20 copepods per individual, suggesting an impact of parasite dose-dependent infection probability for *Dracunculus* infection in paratenic hosts. As copepod infection rate during this study was estimated to be $\ge$ 25%, it is likely that animals ingesting 20 copepods would consume at least 5 *Dracunculus* sp. larvae. Previous studies demonstrated that 10 larvae (administered IP) were sufficient to infect a ferret, but that percent recovery was higher with IP infection than PO^27,28^. It is likely that a similar minimum infectious dose also exists for paratenic hosts and may differ by paratenic host species and mode of infection. Parasite dose-dependent infection probability of *Dracunculus* spp. merits further investigation, as understanding this relationship could help researchers to more effectively study transmission in the laboratory by performing experimental infection trials with greater reliability.

Despite the variable sample sizes and exposure routes in this study, we demonstrated that a wide range of animals (anurans, fish, and lizards) were susceptible to infection with *D. insignis* and/or *D. medinensis* L3s. Importantly, one exposed fish species (*Synodontis eupterus*) was susceptible, opening up further concerns that certain fish species could serve as transport and paratenic hosts of *Dracunculus* species. Nile monitor lizards and anoles were successfully infected with L3s, demonstrating the first experimental infection of lizards with *Dracunculus* larvae. *Dracunculus* larvae remained L3s in the tissues of tested anurans for up to 244 days, extending the known persistence time of infectious larvae. Although no larvae were recovered from frogs that were fed L3s recovered from other paratenic hosts, continued investigation into the possibility of paratenic host to paratenic host transmission would be particularly interesting in determining if some predatory frogs (tadpoles or adults), fish, or lizards may concentrate higher numbers of L3s over time through predation of other infected paratenic hosts. Despite this study not determining how infectious larvae recovered from each of these paratenic hosts would be to another host, our findings contribute to a better understanding of the ability of these paratenic hosts to harbor *Dracunculus* L3s. This information is valuable to understanding how transmission to animal definitive hosts may be occurring, in addition to informing GWEP management decisions aiming to decrease transmission of *D. medinensis* to humans and animals.

## Methods

### Copepods and *Dracunculus* larvae

*Dracunculus insignis* larvae used in this study were obtained from raccoons from Georgia, USA in April and May of 2016^19^. *Dracunculus medinensis* larvae were obtained from infected dogs in Guinea worm endemic zones along the Chari River in Chad, Africa in May through July of 2016. Lab-raised copepods (from wild-caught stock from Athens, Georgia, USA) of the genus *Macrocyclops* were used for *D. insignis* trials and wild-caught African copepods most similar to the genus *Mesocyclops* were used for *D. medinensis* trials. Copepod identification was confirmed by sequence analysis of the partial cytochrome oxidase subunit 1 (COI) gene (Genbank accession numbers: MW522586 and MW522587)^29^. Copepods were infected by adding *Dracunculus* L1s to their water in a 1:3 ratio and allowing copepods to feed for 72 h. After two weeks, copepods were checked via microscopy to ensure *Dracunculus* larvae had molted to L3s. Copepod infection rate and *Dracunculus* development was assessed as previously described^7^. Batches of copepods with $\ge$ 25% infection rate (most often one larva per infected copepod) were used in trials.

### Animals used in this study

Aquatic or semi-aquatic animals that may be prey for definitive hosts of *Dracunculus* spp. were chosen for their suspected involvement in transmission. African species were chosen primarily for their potential to transmit *D. medinensis* and North American species were chosen for their potential to transmit *D. insignis* and other North American *Dracunculus* species. Five species of anurans were used. African clawed frogs were captive-bred (Xenopus Express, Brooksville, Florida, USA). All other anuran species were collected from the wild in Georgia, USA (American bullfrog [*Lithobates* (*Rana*) *catesbeianus*], American toad, Cope’s gray treefrog, and southern leopard frog). Tadpoles were also identified to species^30^. Anurans were assigned to age groups based on Gosner stage: tadpoles were categorized as Gosner stages 26–42, froglets were categorized as Gosner stages 43–45, and adults were Gosner stage 46 and older. The lizards used were juvenile, captive-bred Nile monitor lizards (Backwater Reptiles, Rocklin, California, USA) and adult, wild-caught green anoles from Georgia, USA. Two species of captive-bred, commercially sourced fish, bichir and featherfin catfish, were included in the study.

### Infection methods

Animals were exposed by one of two methods: group batch or PO (Tables 1, 2). Animals that were exposed via group batch were added to a 500 ml beaker (n = 1–8) containing infected copepods and allowed to feed up to 72 h, until all copepods were ingested. For those species that did not readily consume copepods or were too large to expose in a small beaker, infection PO was performed by concentrating infected copepods in a small volume of water and performing oral gavage with a pipette. The pipette was rinsed to ensure all copepods were ingested. If copepods were recovered during the rinse, oral gavage was repeated until all copepods were ingested.

### Infection of potential paratenic hosts with *Dracunculus*-infected copepods

The number of animals exposed to *Dracunculus* larvae depended on the availability of infectious larvae and infected copepods. Fifty-nine individual anurans of four species were exposed to *D. insignis*-infected copepods via group batch (Table 1). Thirty-seven anurans were exposed in batches with 10 *D. insignis*-infected copepods per individual; 22 anurans were exposed in batches with 20 *D. insignis-*infected copepods per individual (Table 1). Seven anurans, all adults, were exposed PO to 20 *D. medinensis*-infected copepods (Table 1). Five individual Nile monitor lizards were exposed PO to *D. insignis*-infected copepods (Table 1). The single green anole was exposed PO with both *D. insignis-* and *D. medinensis*-infected copepods (Table 1). Eight individual fish (four bichir and four featherfin catfish) were exposed to *D. medinensis* larvae PO or by group batch methods with infected copepods (Table 1). Animals were euthanized after a varying number of days post exposure (6–140) and examined for the presence of *Dracunculus* larvae (Table 1).

### Ability of *Dracunculus* L3s from a paratenic host to infect another paratenic host

Some *D. medinensis* L3s recovered from experimentally infected animals (*Hyla chrysoscelis* and *Synodontis eupterus* (Table 1)) were administered PO to two adult African clawed frogs (11 and 15 larvae were given to each animal, respectively). These two African clawed frogs were euthanized four months post-inoculation and examined for the presence of *Dracunculus* larvae.

### Persistence of *Dracunculus* in paratenic hosts

Thirty-one adult African clawed frogs were exposed PO to 50 *D. insignis-* or *D. medinensis*-infected copepods (Table 2). Frogs were euthanized at approximately two-, three-, four-, five-, six-, and eight-months post-exposure (Table 2).

### Recovery of *Dracunculus* larvae from paratenic hosts

Animals used in this study were humanely euthanized using a buffered MS-222 bath (anurans and fish) or isoflurane inhalation (lizards) followed by pithing and cervical dislocation^31^. Gastrointestinal tract and muscle tissues were placed in separate Petri dishes, macerated, and allowed to sit at room temperature in water. Petri dishes were observed under a dissecting microscope for movement of larvae immediately after necropsy and tissue preparation and then again at four, eight, and 24 h. For larger animals (i.e., fish, adult African clawed frogs, and lizards), tissues were divided into multiple dishes.

### Ethical approval and informed consent

All animal procedures in this study were reviewed and approved by the University of Georgia Institutional Animal Care and Use Committee (A2018 01-010). Additionally, all methods performed were in accordance with ARRIVE guidelines and within the aforementioned approved animal use protocol.

## Data Availability

All data generated or analyzed during this study are included in this published article.
